# Platelet-Rich Fibrin Decreases the Inflammatory Response of Mesenchymal Cells

**DOI:** 10.3390/ijms222111333

**Published:** 2021-10-20

**Authors:** Zahra Kargarpour, Jila Nasirzade, Layla Panahipour, Richard J. Miron, Reinhard Gruber

**Affiliations:** 1 Department of Oral Biology, Medical University of Vienna, 1090 Vienna, Austria; zahra.kargarpooresfahani@meduniwien.ac.at (Z.K.); jila.nasirzaderajiri@meduniwien.ac.at (J.N.); layla.panahipour@meduniwien.ac.at (L.P.); 2 Department of Periodontology, School of Dental Medicine, University of Bern, 3010 Bern, Switzerland; richard.miron@zmk.unibe.ch

**Keywords:** platelet-rich fibrin, inflammation, Toll-like receptor, mesenchymal cells, cytokine

## Abstract

Chronic inflammation is a pathological process where cells of the mesenchymal lineage become a major source of inflammatory mediators. Platelet-rich fibrin (PRF) has been shown to possess potent anti-inflammatory activity in macrophages, but its impact on mesenchymal cells has not been investigated. The aim of this study was, therefore, to expose mesenchymal cells to inflammatory cytokines together with lysates generated from liquid platelet-poor plasma (PPP), the cell-rich buffy coat layer (BC; concentrated-PRF or C-PRF), and the remaining red clot layer (RC), following centrifugation of blood. Heating PPP generates an albumin gel (Alb-gel) that when mixed back with C-PRF produces Alb-PRF. Membranes prepared from solid PRF were also subjected to lysis. We report here that lysates of PPP, BC, and PRF decreased the cytokine-induced expression of interleukin 6 (IL6) and nitric oxide synthase (iNOS) in the bone marrow-derived ST2 cells. Consistently, PPP, BC, and PRF greatly decreased the phosphorylation and nuclear translocation of p65 in ST2 cells. The inflammatory response caused by Pam3CSK4 was reduced accordingly. Moreover, PPP, BC, and PRF reduced the enhanced expression of inflammatory mediators IL6 and iNOS in 3T3-L1 pre-adipocyte mesenchymal cells, and iNOS and CCL5 in murine calvarial cells. Surprisingly, PRF lysates were not effective in reducing the inflammatory response of human gingival fibroblasts and HSC2 epithelial cells. The data from the present study suggest that both liquid PRF and solid PRF exert potent anti-inflammatory activity in murine mesenchymal cells.

## 1. Introduction

Chronic inflammatory diseases share a conserved pathological mechanism where the immune response fails to remove the detrimental agents, and thus the resolution of inflammation is hampered [1,2,3]. Chronic inflammation drives a catabolic process that culminates in tissue destruction [4,5]. This catabolic process is not restricted to the periodontium, and when not resolved, follows a similar mechanism of destruction as found in rheumatoid arthritis [6], Crohn’s disease [7], psoriasis [8], and refractory leg ulcers [9]. The detrimental agents are manifold and often use the innate immunity trigged by the activation of pattern recognition receptors expressed on macrophages. Activated macrophages release a large spectrum of pro-inflammatory cytokines such as IL1β, IL6, IL17, and TNFα [2,10]. The pro-inflammatory cytokines initiate a feed-forward process whereby neighboring cells are forced to produce pro-inflammatory cytokines and chemokines. These neighboring cells can be of the mesenchymal lineage, including fibroblasts of the soft connective tissue [11]. Chronic inflammation not only causes tissue destruction but also undermines the cellular process of tissue regeneration [6]. There is thus a great demand on local strategies to dampen the inflammatory process with an emphasis on cells of the mesenchymal lineage.

Platelet-rich fibrin (PRF), a concentrate of cells and growth factors generated from the centrifugation of whole blood, is one local technique aimed at assisting in alveolar ridge preservation [12,13,14], increasing the width of the keratinized mucosa [15], treatment of periodontal intrabony defects [16], and treating gingival recessions [17]. Moreover, PRF is effective in the treatment of ulcers [9] and is used in other fields such as facial esthetics [18,19] and sports medicine [19], including meniscal repair [20]. The clinical effect of PRF can partially be explained by its growth factors, which can be identified by proteomics analysis [21,22] and immunoassays [23,24]. In vitro, PRF causes an M1-to-M2 transition in macrophage polarization [25,26] and reduces osteoclastogenesis [26,27]. Injectable PRF was shown to have an anti-inflammatory effect on macrophages and dendritic cells [28]. Recently, L-PRF was reported to suppress LPS-induced inflammatory responses in Schwann cells [29] and lower the inflammatory response of gingival fibroblasts to LPS in vitro [30], inspiring further research towards the various fractions and preparation of PRF.

PRF is easily accessible and, apart from the investment into a centrifuge and consumables, is a rather low-cost treatment option. Nevertheless, there is an ongoing debate on the ideal protocol for PRF preparation with respect to the relative centrifugation force, the time of centrifugation, and the use of horizontal versus fixed angle centrifuges [31,32]. The debate also includes possible unwanted side effects of the silicone and silica particles found on blood collection tubes to prepare solid PRF [33,34]. This can be entirely avoided when liquid PRF is produced in chemical-free plastic tubes. Platelets and leucocytes accumulate at the interface towards the red clot in a zone termed the “buffy coat” [35,36]. Moreover, the platelet-poor plasma (PPP) layer above the buffy coat can be subjected to heat treatment so that the albumin fraction is coagulated, thereby forming an “albumin gel” that maintains stability for months in vivo [35,36]. We have recently shown that it is in particular the PPP, BC, and PRF lysates that greatly suppress the inflammatory response of macrophages in vitro [26]. However, their respective effects on cells of the mesenchymal lineage are not fully understood.

Mesenchymal cells can maintain their potential to differentiate into tissues of mesenchymal origin including bone, cartilage, and adipose tissue [37]. Fibroblasts of the gingiva or the skin are also of mesenchymal origin [38]. Cell lines represent at least some of the major characteristics of primary cells. The bone marrow-derived mesenchymal cell line ST2 can undergo osteogenic and adipogenic differentiation in vitro [39,40]. ST2 cells increase IL6 expression in response to prostaglandin E_2_ exposure [41]. The 3T3-L1 pre-adipocyte mesenchymal cells are an established model for adipogenesis, also used for inflammation research [42]. Gingival fibroblasts are a widely used model for the investigation of inflammation of the oral cavity and oral disease [43] and have been utilized in PRF research [44,45]. The goal of the present study was to take advantage of the ST2 and 3T3-L1 cells and gingival fibroblasts to study the possible anti-inflammatory activity of the various PRF preparations in vitro.

## 2. Results

### 2.1. PPP, BC, and PRF Reduced Cytokine-Induced Inflammation in Murine Mesenchymal Cells

**Figure 1:**
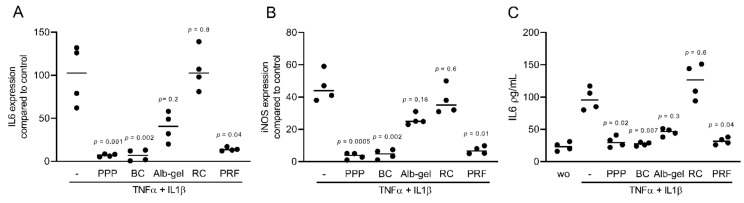
PPP, BC, and PRF lysates reduced cytokine-induced inflammation in murine ST2 cells. ST2 cells were treated with 10% of PPP, BC, Alb-gel, RC, and 30% of PRF in the presence of the inflammatory cytokines TNFα and IL1β. (**A**,**B**) Data indicate the x-fold changes of IL6 and iNOS gene expression (**C**) and the IL6 levels in the cell supernatant, n = 4. WO means without and represents unstimulated cells.

**Figure 2:**
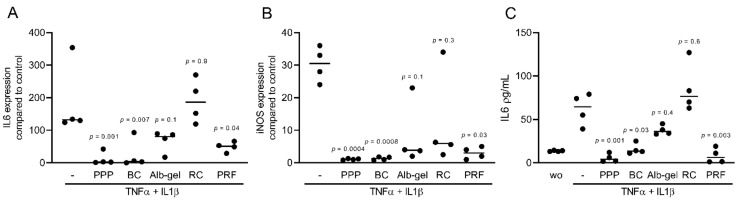
PPP, BC, and PRF lysates reduced cytokine-induced inflammation in murine 3T3-L1 cells. 3T3-L1 cells were treated with 10% of PPP, BC, Alb-gel, RC, and 30% of PRF in the presence of the inflammatory cytokines TNFα and IL1β. (**A**,**B**) Data indicate the x-fold changes of IL6 and iNOS expression (**C**) and the IL6 levels in the cell supernatant, n = 4.

**Figure 3:**
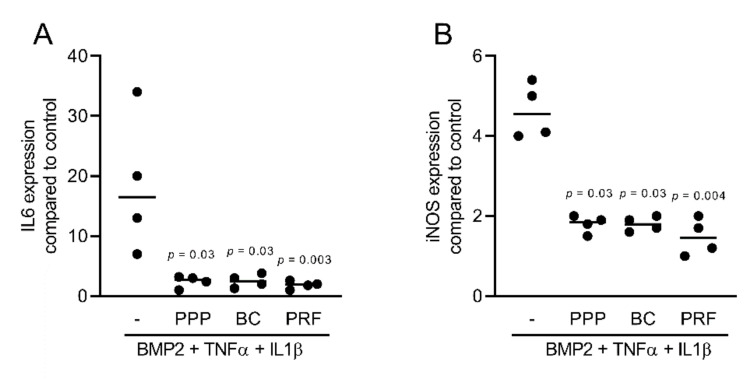
PPP, BC, and PRF lysates reduced inflammation in murine ST2 cells preincubated by BMP2. ST2 cells were treated with BMP2 for 48 h, followed by addition of 10% PPP, BC, and 30% PRF in the presence of the inflammatory cytokines TNFα and IL1β. (**A**,**B**) Data indicate the x-fold changes of IL6 and iNOS gene expression, n = 4.

**Figure 4:**
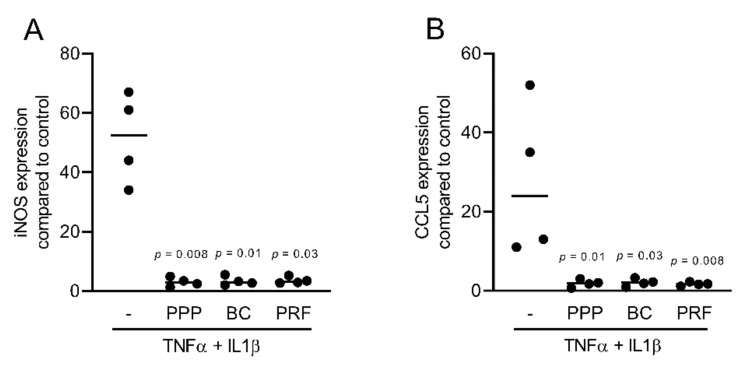
PPP, BC, and PRF lysates reduced inflammation in murine calvaria-derived cells. Calvaria cells were exposed to 10% of PPP, BC, and 30% of PRF in the presence of the inflammatory cytokines TNFα and IL1β. (**A**,**B**) Data indicate the x-fold changes of iNOS and CCL5 gene expression, n = 4.

**Figure 5:**
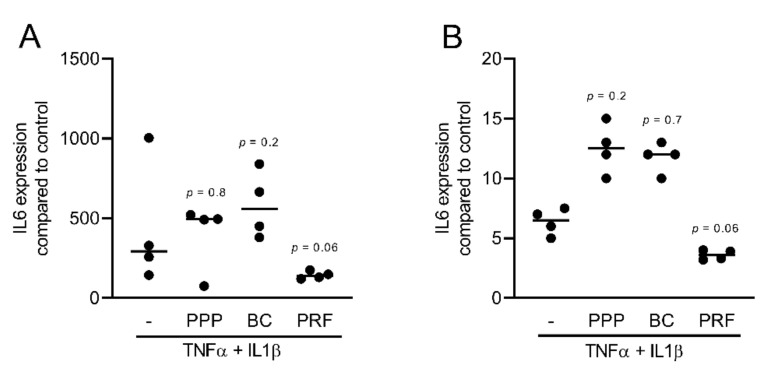
None of the fractions reduced inflammation in gingival fibroblasts and HSC2. Gingival fibroblasts and HSC2 were incubated with 10% of PPP, BC, and 30% of PRF in the presence of TNFα and IL1β. In gingival fibroblasts, 10% of Alb-gel and RC were also added. (**A**,**B**) Data indicate the x-fold changes of IL6 gene expression, n = 4.

To assess the anti-inflammatory effects of different fractions of PRF, we exposed murine ST2, 3T3-L1, and calvaria cells along with human gingival fibroblast and HSC2 cells to TNFα and IL1β, either with or without 10% of PPP, BC, Alb-gel, RC, and 30% of PRF lysates [25,26]. Gene expression analysis showed that PPP, BC, and PRF lysates notably reduced the inflammatory response in ST2 and 3T3-L1 cells. Alb-gel and RC fractions, however, failed to significantly reduce the expression of IL6 and iNOS genes (Figure 1A,B and Figure 2A,B). To understand if cells with a more osteogenic phenotype are also susceptive to PRF lysates, we preexposed ST2 cells to the strong osteogenic differentiation factor BMP2 for 48 h [21]. Gene expression analysis showed that PPP, BC, and PRF lysates are all capable of decreasing IL6 and iNOS under these conditions (Figure 3A,B). To confirm these findings obtained by gene expression analysis, we measured levels of IL6 protein in the supernatants. Consistently, PPP, BC, and PRF lysates, but not Alb-gel and RC, reduced the production of IL6 (Figure 1C and Figure 2C). Moreover, in primary calvaria cells, PPP, BC, and PRF lysates greatly dampened the expression of iNOS and CCL5 (Figure 4A,B). IL6 was not considerably reduced, neither by PCR nor by ELISA (data not shown). In gingival fibroblasts and HSC2, none of the fractions could significantly reduce the inflammatory response indicated by expression of IL6 (Figure 5A,B) or IL8 (Figure S1).

### 2.2. PPP, BC, and PRF Can Suppress NF-κB Signalling

**Figure 6:**
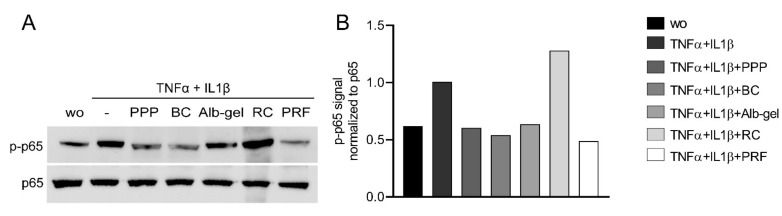
PPP, BC, and PRF could weaken phosphorylation of p65 in ST2 cells. Western blot analysis was carried out for phospho-p65 and total p65. (**A**) ST2 cells were treated with TNFα and IL1β in the presence or absence of 10% of PPP, BC, Alb-gel, RC, and 30% of PRF lysates. (**B**) Data indicate the relative changes normalized to p65.

**Figure 7:**
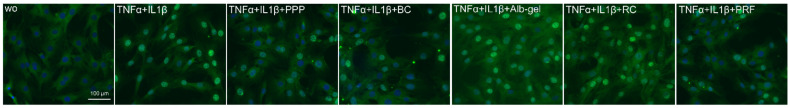
PPP, BC, and PRF attenuated the translocation of NF-κB from the cytoplasm into the nucleus. ST2 cells were exposed to TNFα and IL1β with or without PPP, BC, Alb-gel, RC, and PRF. Immunofluorescence analysis of intracellular translocation of NF-κB p65 into the nucleus. Blue nuclei indicate the unstained cells and green nuclei are positive stained cells. WO means without and represents unstimulated cells.

To further confirmed the ability of PPP, BC, and PRF to attenuate NF-κB p65 signaling, we carried out Western blot analysis. ST2 cells were treated with 10% PPP, BC, Alb-gel, RC, and 30% PRF lysates for 30 min, followed by exposure to TNFα and IL1β for another 30 min. PPP, BC, and PRF suppressed phosphorylation of p65 in ST2 cells (Figure 6A,B). To further evaluate the inhibitory effect of PPP and BC on inflammation, we performed an immunofluorescent analysis of NF-κB translocation. PPP, BC, and PRF strongly reduced the NF-κB p65 translocation induced by TNFα and IL1β in ST2 cells (Figure 7). The respective percentage of nuclear staining was calculated (Figure S2). This finding suggested that PRF can attenuate NF-κB p65 signaling in ST2 cells.

### 2.3. PPP and BC Can Suppress TLR2-Induced Inflammation in ST2 Cells

**Figure 8:**
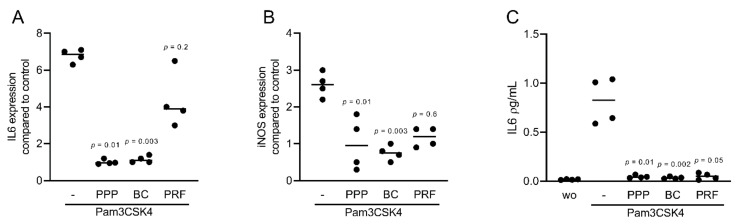
PPP, BC, and PRF can reduce inflammation provoked by TLR2 agonist in ST2 cells. The ST2 cells were exposed to 10% PPP, BC, and 30% PRF lysates in the presence of 5 μg/mL Pam3CSK4, agonists of TLR2. (**A**,**B**) Data show the x-fold changes of IL6 and iNOS gene expression, and (**C**) the concentration of IL6 in the supernatant of ST2 cells. n = 4.

To confirm the findings observed for the cytokine-induced inflammation, we exposed ST2 cells to Pam3CSK4, the agonist of TLR2 in the presence or absence of 10% PPP, BC, and 30% of PRF lysates. Lysates of all preparations suppressed the rather weak inflammatory response provoked by Pam3CSK4 in ST2 cells (Figure 8A,B). Notably, all preparations substantially reduced the IL6 levels (Figure 8C). Altogether, these results suggest that the anti-inflammatory effects of PPP, BC, and PRF can be extended towards TLR agonists in murine ST2 cells.

## 3. Discussion

Since the clinical introduction of PRF in 2006, numerous attempts were made to parallel the clinical research by deciphering possible cellular and molecular mechanisms that explain the promising clinical observations [46]. Today’s basic research mainly focuses on how PRF affects the proliferation and migration of cells of the mesenchymal lineage [47] and the impact of PRF on gene expression driven by TGF-β [21,27,45,47]. Studies investigating the impact of PRF on inflammation have been restricted to cells of the hematopoietic lineage, e.g., macrophages [25,26,28] and dendritic cells [28]. This evidence suggests that PRF is capable of shifting the pro-inflammatory M1 towards a resolving M2 phenotype [25,26,28]. Recently, L-PRF was reported to exert anti-inflammatory properties in LPS-treated gingival fibroblasts [30]. Nevertheless, the question as to whether or not solid and liquid PRF exert an anti-inflammatory activity in cells of the mesenchymal lineage has not yet been fully elucidated. Moreover, the PRF fractions have not been tested. In the present study, we clearly show the anti-inflammatory activity of the various fractions and preparations of liquid and solid PRF in murine cells of the mesenchymal lineage.

The first main finding was that in accordance with our previous study on murine macrophages [26], not only BC and PPP possess an anti-inflammatory activity in ST2 and 3T3-L1 cells, but also PRF lysates caused a robust inhibition of the inflammatory response to TNFα and IL1β. This activity was independent of BMP-2, a strong inducer of osteogenic differentiation [21]. Moreover, reduction of p65 phosphorylation and nuclear translocation suggests an attenuated NF-κB signaling caused by PPP, BC, and PRF lysates. When ST2 cells were exposed to Pam3CSK4, the inflammatory response was rather weak, but nevertheless, PPP, BC, and PRF lysates exerted their anti-inflammatory activity by reducing IL6 and iNOS expression. Further confirmation was derived from experiments using murine calvaria cells; however, while PPP, BC, and PRF greatly reduced iNOS and CCL5, no considerable reduction in IL6 production was observed. Taken together, the anti-inflammatory activity of the blood fractions was not restricted to TNFα and IL1β but extended towards the TLR signaling pathway. It would now be interesting to know which blood-derived molecule is responsible for the anti-inflammatory activity of PPP, BC, and PRF lysates in murine macrophages [25,26] and the respective mesenchymal cells.

The second main result was that the expected decrease of IL6 and IL8 by PRF was not observed in human gingival fibroblast or the HSC2 oral squamous cell carcinoma cell line. Knowing that the cell source is important [48], there is obviously a difference in the responsiveness of murine and human cells to PPP, BC, and PRF lysates with respect to the anti-inflammatory activity in vitro. This was unexpected as L-PRF suppressed the LPS-induced inflammatory responses in gingival fibroblasts [30]. In the present study, however, the origin of the blood fractions is human, and the effects were restricted to the murine cells. The findings that murine but not human cell of the mesenchymal lineage were susceptible to PRF leaves room for speculations. We used culture-expanded gingival fibroblasts, which can undergo cellular aging and senescence [49]. Aging and senescence might affect the responsiveness of the gingival fibroblast cells to PRF, but this is unlikely because the cells showed a robust response to TNFα and IL1β. Moreover, we used an allogenic and xenogenic model; donors of PRF and fibroblasts are not identical and were even tested with murine cells. Allogenicity is critical when the immune system recognizes foreign molecules upon organ donation [50]. However, when considering the blood group and rhesus factor, research has found that blood transfusion is of low risk for the patient [51]. Moreover, mesenchymal cells are not classical immune cells; thus, graft versus host reactions can be ruled out. Mice’s blood volume is also too low to generate PRF for in vitro testing. Another potential limitation is that we have not tested platelet-rich plasma (PRP), the first generation of platelet concentrates prepared from anticoagulated blood [52]. Overall, the in vitro setting seems acceptable. Nevertheless, we need to understand why the anti-inflammatory activity blood-derived molecules are restricted to mesenchymal cell of murine origin.

The study has more limitations. While the role of heating PPP and its effect on anti-inflammatory activity is obvious, the impact of lysates prepared from the remaining red clot remains enigmatic. The data leave us with the impression that the red lysates occasionally enhance the already strong inflammation signal induced by TNFα and IL1β in ST2 and 3T3-L1 cells. Our observation suggests that further studies need to be conducted to elucidate whether or not the red cells can intensify inflammation. This is a reasonable assumption since the red blood cells from healthy individuals regulate proliferation and activity of T cells through modulating cytokine interactions [53] and they serve as a cytokine reservoir [54]. Future studies discovering the inflammatory role of erythrocytes in the blood fractions are proposed. The knowledge acquired from comparing the in vitro effects of a natural blood clot to PRF membranes will aid in the resolution of this problem. Finally, another study limitation is that we can only speculate about the clinical relevance of our findings. The pro-resolving PRF might generate a local milieu that supports bone formation to occur. The clinical implications of the current findings should, however, be interpreted carefully particularly because we could not show the anti-inflammatory effects of PRF in human cells.

Taken together, we report here that it is not only the cell-rich BC but also the PPP and the PRF lysates derived from the PRF membrane that hold a potent anti-inflammatory activity in murine mesenchymal cells. The same effect was not observed in the mesenchymal cells of human origin, which emphasizes the importance of the cell source and the species.

## 4. Materials and Methods

### 4.1. Isolation and Culture of Murine ST2, 3T3-L1, and Calvaria Cells, and Human Gingival Fibroblasts and HSC2

The ST2 mesenchymal stromal cells were isolated from mouse bone marrow (RIKEN Cell Bank, Tsukuba, Japan), 3T3-L1 murine preadipocyte cell line kindly was donated by Christian Wolfrum (ETH Zürich, Switzerland), and the oral squamous cell carcinoma cell line HSC2 was obtained from Health Science Research Resources Bank (Sennan, Japan). The cells were expanded in growth Dulbecco’s modified Eagle’s medium (DMEM, Sigma-Aldrich, St. Louis, MO, USA), 10% fetal calf serum (Bio&Sell GmbH, Nuremberg, Germany), and 1% antibiotics (Sigma Aldrich, St. Louis, MO, USA) and seeded at 3 × 10^5^ cells/cm^2^ into 24-well plates. Human gingiva was harvested from extracted wisdom teeth from patients who had given informed and written consent. An approval was obtained from the Ethics Committee of the Medical University of Vienna (EK NR 631/2007). A total of three strains of fibroblasts were generated by explant cultures, and fewer than five passages were used for the experiments. Gingival fibroblasts were also expanded in the growth medium. To prepare calvaria cells, we euthanized mouse pups less than 5 days old. Organ donation from mice required an informal approval of the local veterinarian authorities but not a formal approval by the Ethics Committee according to Austrian law. Murine calvaria was subjected to 0.1% collagenase I and 0.2% dispase (both Gibco, Life Technologies Corp., Thermo Fisher Scientific, Waltham, MA, USA) sequential digestion. The first digest was discarded, and the subsequent digests were pooled and expanded before freezing. For each experiment, cells were seeded at a concentration of 3 × 10^5^ cells/cm^2^ onto culture dishes one day prior to stimulation. Cells were treated overnight with and without different 10% of PPP and BC and 30% of PRF lysates in serum-free media under standard conditions at 37 °C, 5% CO_2_, and 95% humidity. This setting was performed with and without TNFα and IL1β at 20 ng/mL (both Sigma Aldrich, St. Louis, MO, USA) or Pam3CSK4 at 5 µg/mL (InvivoGen, Toulouse, France) to induce an inflammatory response. In indicated experiments, ST2 cells were exposed to 300 ng/mL recombinant BMP-2 (ProSpec-Tany TechnoGene Ltd., Rehovot, Israel) for 48 h prior to provoking the inflammatory response.

### 4.2. Preparation of PPP, Buffy Coat, Red Clot, and PRF Lysates

Volunteers signed informed consent, and the ethics committee of the Medical University of Vienna (1644/2018) approved the preparation of PRF. All experiments were performed in accordance with relevant guidelines, and regulations and were conducted in accordance with the Declaration of Helsinki (1975), as revised in 2013. For preparing liquid PRF fractions from non-coagulated blood, we collected venous blood from healthy volunteers, three females and three males from 23 to 35 years, in plastic tubes (“No Additive“, Greiner Bio-One GmbH, Kremsmünster, Austria) and centrifuged it at 2000× *g* for 8 min (swing-out rotor; Z306 Hermle, Universal Centrifuge, Wehingen, Germany). The uppermost 2 mL PPP, the 1 mL buffy coat (C-PRF), and the 1 mL erythrocyte fraction next to BC/C-PRF were collected. To generate Alb-gels, we immediately heated PPP at 75 °C for 10 min (Eppendorf, Thermomixer F1.5, Hamburg, Germany) before it was placed on ice. Every 1 mL fraction of the solid Alb-gel was then transferred into 1 mL of serum-free media. PRF membranes from coagulated blood were produced using glass tubes with no silica/silicon added (Bio-PRF, Venice, FL, USA) with centrifugation at 1570 RPM for 12 min (RCF-max = 400 g). PRF membranes were produced using a centrifuge device with universal swing-out rotors (Z306 Hermle, Universal Centrifuge, Wehingen, Germany). The PRF clot was separated from the remaining red thrombus and compressed between two layers of dry gauze. Thereafter, each PRF membrane was transferred into serum-free medium (1 cm PRF/mL). All the blood fractions and the Alb-gel were subjected to two cycles of freeze–thawing followed by sonication (Sonopuls 2000.2, Bandelin electronic, Berlin, Germany). After centrifugation (Eppendorf, Hamburg, Germany) at 15,000× *g* for 10 min, 1 mL aliquots of lysates were stored at −20 °C for no longer than one month. The lysates were thawed, and cells were exposed as indicated above.

### 4.3. Reverse Transcription Quantitative Real-Time PCR (RT-qPCR) and Immunoassay

For RT-qPCR [55], after overnight stimulation, total RNA was isolated with the ExtractMe total RNA kit (Blirt S.A., Gdańsk, Poland) followed by reverse transcription (LabQ, Labconsulting, Vienna, Austria) and polymerase chain reaction (LabQ, Labconsulting, Vienna, Austria) on a CFX Connect™ Real-Time PCR Detection System (Bio-Rad Laboratories, Hercules, CA). Primer sequences were mIL6-F: GCTACCAAACTGGATATAATCAGGA, mIL6-R: CCAGGTAGCTATGGTACTCCAGAA; mGAPDH-F: AACTTTGGCATTGTGGAAGG, mGAPDH-R: GGATGCAGGGATGATGTTCT; miNOS-F: GGTGAAGGGACTGAGCTGTT, miNOS-R: ACGTTCTCCGTTCTCTTGCAG; mCCL5-F: CCTGCTGCTTTGCCTACCTC, mCCL5-R: ACACACTTGGCGGTTCCTTC; hIL6-F: GAAAGGAGACATGTAACAAGAGT, hIL6-R: GATTTTCACCAGGCAAGTCT; hIL8-F: AACTTCTCCACAACCCTCTG, hIL8-R: TTGGCAGCCTTCCTGATTTC; hGAPDH-F: AAGCCACATCGCTCAGACAC, hGAPDH-R: GCCCAATACGACCAAATCC. The mRNA levels were calculated by normalizing to the housekeeping gene GAPDH using the ΔΔCt method. Supernatants were analyzed for IL6 secretion by immunoassay according to the manufacturer’s instruction (R&D Systems, Minneapolis, MN, USA). RT-PCR data are represented in comparison with the untreated control, which was considered 1.0 in all the measurements, and therefore there was no need to show it as a separate group. However, in IL6 ELISA, the absolute amount of secreted protein from the cells was reported, and therefore untreated cells were also considered to show the amount of protein in all the samples and compare the protein concentration.

### 4.4. Immunofluorescence

The immunofluorescent analysis of p65 nuclear translocation was performed in ST2 cells plated onto Millicell^®^ EZ slides (Merck KGaA, Darmstadt, Germany) at 1.5 × 10^5^ cells/cm^2^. Cells were exposed to 10% of PPP, BC, Alb-gel, and RC or 30% of PRF lysates for 30 min following overnight serum starvation. Thereafter, the cells were exposed to TNFα and IL1β for another 30 min. The cells were fixed with 4% paraformaldehyde, blocked with 1% bovine serum albumin (Sigma Aldrich, St. Louis, MO, USA), and permeabilized with 0.3% TritonX-100 (Sigma Aldrich, St. Louis, MO, USA). We used NF-κB p65 antibody (anti-rabbit IgG, 1:800, Cell Signaling Technology, Cambridge, United Kingdom) at 4 °C overnight. Detection was with the goat anti-rabbit Alexa 488 secondary antibody (CS-4412, 1:1000, Cell Signaling Technology). Images were captured under a fluorescent microscope with a dual excitation filter block DAPI-FITC (Echorevolve Fluorescence microscope, San Diego, CA, USA). The number of blue nuclei to green nuclei were calculated.

### 4.5. Western Blot

ST2 cells were seeded at 5 × 10^5^ cells/cm^2^ into 12-well plates. The following day, serum-starved cells were exposed to 10% of PPP, BC, Alb-gel, and RC or 30% of PRF lysates for 30 min, and then they were exposed to TNFα and IL1β for another 30 min. Extracts containing SDS buffer with protease and phosphatase inhibitors (cOmplete ULTRA Tablets and PhosSTOP; Roche, Mannheim, Germany) were separated by SDS-PAGE and transferred onto PVDF membranes (Roche Diagnostics, Mannheim, Germany). Membranes were blocked, and the binding of the first antibody phospho-NF-κB p65 antibodies (anti-rabbit IgG, 1:1000, Cell Signaling Technology) and NF-κB p65 antibody (1:1000, Cell Signaling Technology) was detected with the second antibody labelled with HRP (CS-7074, anti-rabbit IgG, 1:10,000, Cell Signaling Technology). After their exposure to the Clarity Western ECL Substrate (Bio-Rad Laboratories, Inc., Hercules, CA, USA), chemiluminescence signals were visualized with the ChemiDoc imaging system (Bio-Rad Laboratories). For densitometric analysis of blots, the WB images were analyzed using Image Lab software (Bio-Rad Laboratories).

### 4.6. Statistical Analysis

All experiments were performed four times. Statistical analysis of the IL6 and iNOS expression and immunoassay for IL6 was performed with the Kruskal–Wallis test for multiple comparison without correction of *p*-values. The results for the treatment groups were compared with TNFα and IL1β, or Pam3CSK4 group as the positive control. Analyses were performed using Prism v8 (GraphPad Software, La Jolla, CA, USA). Significance was set at *p* < 0.05.

## Data Availability

The original contributions presented in the study are included in the article/supplementary material. Further inquiries can be directed to the corresponding author.

## Supplementary Materials

The following are available online at https://www.mdpi.com/article/10.3390/ijms222111333/s1.
