# Supplementary material for: Platelet-Rich Fibrin Decreases the Inflammatory Response of Mesenchymal Cells
Source: Int J Mol Sci. 2021 Oct 20;22(21):11333. doi: 10.3390/ijms222111333 (PMC8583104; doi:10.3390/ijms222111333)
Supplement: Supplementary file 1 [file ijms-22-11333-s001.zip › ijms-1390848-supplementary.pdf]

Supplement Figures:

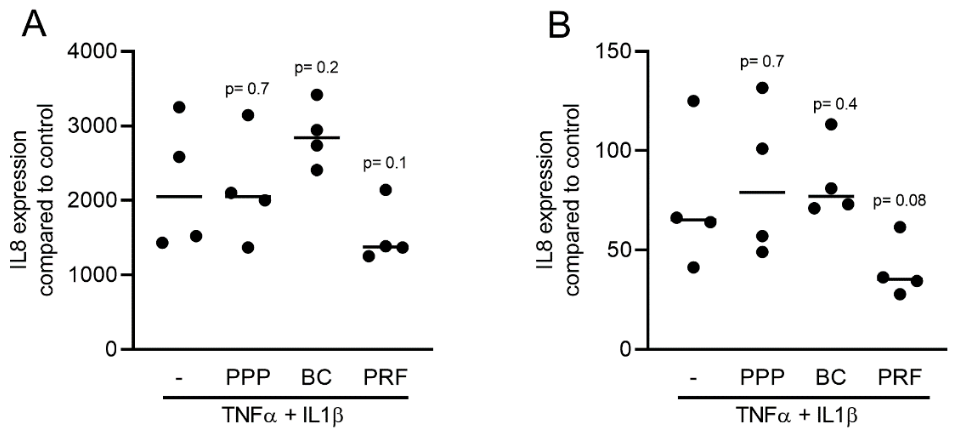

Figure S1. None of the fraction reduced cytokine-induced inflammation in gingival fibroblasts and HSC2.
Gingival fibroblasts and HSC2 were incubated with 10% of PPP, BC and 30% of PRF in the presence of the TNFα and IL1β. Data indicate the x-fold changes of IL8 gene expression in (A) gingival fibroblasts and (B) HSC2 cells, N = 4. Statistical analysis was based on Kruskal Wallis multiple comparison and P values are indicated compared to the positive control.

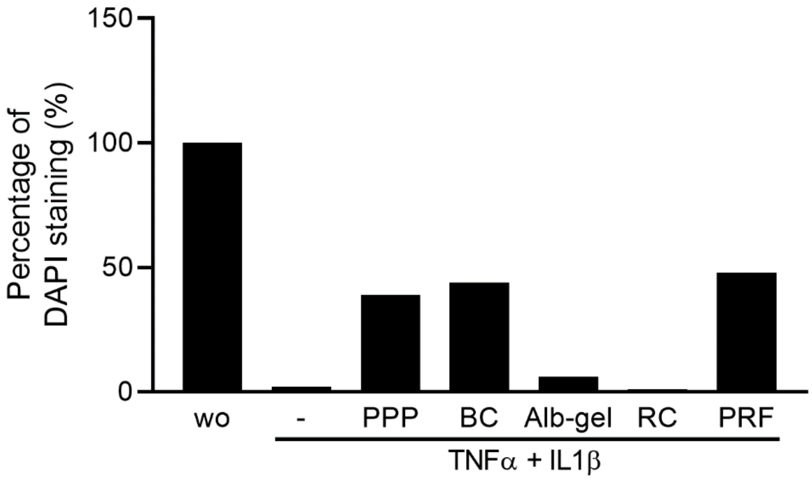

Figure S2. Quantification of nuclear translocation of NFkB.
Counting the number of ST2 cells exposed to TNFα and IL1β with or without PPP, BC, Alb-gel, RC and PRF based on figure 7. Data indicates the percentage of blue nuclei that were exclusively stained with DAPI compared to the total nuclei in each well.
